# Detection of grain yield QTLs in the durum population Lahn/Cham1 tested in contrasting environments

**DOI:** 10.3906/biy-2008-41

**Published:** 2021-02-09

**Authors:** Issame FAROUK, Ahmad ALSALEH, Jihan MOTOWAJ, Fatima GABOUN, Bouchra BELKADI, Abdelkarim FILALI MALTOUF, Zakaria KEHEL, Ismahane ELOUAFI, Nasserelhaq NSARELLAH, Miloudi M. NACHIT

**Affiliations:** 1 Laboratory of Microbiology and Molecular Biology, Department of Biology, Med V University, Rabat Morocco; 2 Department of Science and Technology Bozok University, Yozgat Turkey; 3 ICARDA, The International Center for Agricultural Research in the Dry Areas, Rabat Morocco; 4 INRA, National Institute of Agronomical Research, Unity of Biotechnology Research, Rabat Morocco; 5 ICBA, International Center for Biosaline Agriculture, Dubai United Arab Emirates

**Keywords:** Molecular markers, grain yield, genetic linkage map, SNP, QTL

## Abstract

Durum wheat (*Triticum* *turgidum* L. var durum) is tetraploid wheat (AABB); it is the main source of semolina and other pasta products. Grain yield in wheat is quantitatively inherited and influenced by the environment. The genetic map construction constitutes the essential step in identifying quantitative trait loci (QTLs) linked to complex traits, such as grain yield. The study aimed to construct a genetic linkage map of two parents that are widely grown durum cultivars (Lahn and Cham1) in the Mediterranean basin, which is characterized by varying climate changes. The genetic linkage map of Lahn/Cham1 population consisted of 112 recombinant inbred lines (RILs) and was used to determine QTLs linked to the grain yield in 11 contrasting environments (favorable, cold, dry, and hot). Simple sequence repeat (SSR) molecular markers were used to construct an anchor map, which was later enriched with single nucleotide polymorphisms (SNPs). The map was constructed with 247 SSRs and enriched with 1425 SNPs. The map covered 6122.22 cM. One hundred and twenty-six QTLs were detected on different chromosomes. Chromosomes 2A and 4B harbored the most significant grain yield QTLs. Furthermore, by comparison with several wheat mapping populations, all the A and B chromosomes of Lahn/Cham1 QTLs contributed to grain yield. The results showed that the detected QTLs can be used as a potential candidate for marker-assisted selection in durum breeding programs.

## 1. Introduction

Durum wheat originated through merging of two genomes (AABB) through natural interspecific hybridization and genome duplication (Zhang et al., 2012). Durum wheat (*Triticum* *turgidum* L. var durum) is the primary source of semolina for the production of pasta and couscous in the Mediterranean basin, and its cracked grain for burghul in the Middle East (Elouafi and Nachit, 2004). Moreover, durum wheat is grown mainly in drylands and is affected mainly by year-to-year environmental variation, drought, cold, and heat stresses (Nachit, 1992; El Hassouni et al., 2019).

Grain yield is quantitatively inherited and linked to multiple quantitative trait loci (QTLs) and their interactions with the environment. QTLs are DNA regions associated with a specific trait (Oduola et al., 2003). The phenotyping of grain yield and the use of molecular markers for the genotyping and the construction of a genetic map constitute a crucial step in QTL detection (Heidari et al., 2011). Molecular linkage maps have been constructed for durum wheat by several geneticists (Blanco et al., 1996, 1998; Korzun et al., 1999; Nachit et al., 2001; Elouafi and Nachit, 2004; Hail et al., 2012; Nagel et al., 2014; Alsaleh et al., 2015). The first durum map was made by the interspecific cross and was based on 65 recombinant inbred lines (RILs) and restriction fragment length polymorphism (RFLP) markers (Blanco et al., 1996, 1998), and later simple sequence repeats (SSR) were integrated into this genetic map (Korzun et al., 1999). The second durum map was based on an intraspecific cross Jennah Khetifa/Cham1 with 110 RILS using RFLP, SSR, amplified fragment length polymorphism (AFLP), seed storage proteins, and genes (Nachit et al., 2001). Several other studies on durum genetic mappings were conducted (Maccaferri et al., 2008, 2010; Haile et al., 2012; Dura et al., 2013). Moreover, single nucleotide polymorphisms (SNPs) have been included in durum wheat genetic maps (Poecke et al., 2013).  Some researchers have integrated a significant number of SNP in bread and durum wheat genetic linkage maps (Maccaferri et al., 2015; Su et al., 2018; Zeng et al., 2019). Many QTL studies for different traits have been conducted for wheat (Tixier et al., 1998; Varshney et al., 2000; Sourdille et al., 2003; Elouafi and Nachit, 2004; Li et al., 2007; Pozniak et al., 2007; Maccaferri et al., 2008; Zhang et al., 2008; Bennett et al., 2012; Haile et al., 2012; Zhang et al., 2016; Tura et al., 2019; Xu et al., 2019; Zaïm et al., 2020).

In this study, the Lahn/Cham1 population was phenotyped for grain yield across 11 contrasting environments and was genotyped with mainly SSR and SNP markers to construct the genetic linkage map and to identify the QTLs linked to grain yield. The choice of Lahn and Cham1 as parents was due to their wide cultivation in the Mediterranean basin. Moreover, Lahn is highly productive in favorable environments and Cham1 is widely adapted, yield-stable, and drought-tolerant (Nachit et al., 2001; Kehel et al., 2010; Habash et al., 2014). We have provided the Lahn/Cham1 population to Barcelona University and Tunisia to be phenotyped for stable carbon isotope discrimination and other physiological traits (Bort et al., 2014). Moreover, the current study is the first on the genetic linkage mapping and QTL detection for Lahn/Cham1. (Lahn “Improved durum variety” was wrongly quoted as Jennah Khetifa, landrace in the paper published by Bort et al. (2014)). 

The study’s aims were: 1) to construct and enrich the durum genetic linkage map of the Lahn/Cham1 population with SNP markers, and 2) to determine the QTLs linked to the grain yield in different contrasting environments.

## 2. Materials and methods

### 2.1. Plant materials

The studied durum population consisted of 112 F_12_ lines derived from a single-seed descent selection from the cross ICD-MN91-015 between the varieties Lahn and Cham1. The cross was made at CIMMYT/ICARDA durum breeding program for Mediterranean drylands. Lahn is an improved durum wheat variety developed for favorable and high-input environments with cold tolerance. It is also resistant to yellow rust (*Puccinia striiformis*) and moderately resistant to leaf rust (*Puccinia recondita*), powdery mildew (*Erysiphe graminis*), common bunt (*Tilletia caries*), and to Septoria leaf blotch (*Mycosphaerella graminicola*). Furthermore, Lahn has a large kernel size with superior hectoliter weight, milling extraction index, and gluten strength values. As for Cham1, the second parent of the population, it is also originated from the CIMMYT/ICARDA durum program, adapted to the Mediterranean climate, and has high yield stability. It exhibits an excellent resistance to drought and yellow rust with some tolerance to Russian wheat aphid. Cham1 also has high values for osmotic adjustment and high yellow pigment content, but its gluten strength is weak. However, Cham1 is susceptible to leaf rust, stem rust, *Septoria tritici*, and powdery mildew. This variety has a wide adaptation; it has been released in several countries in the Mediterranean basin and outside (Turkey, Syria, Cyprus, Iraq, Algeria, Portugal, Sudan, etc.) under different names.

### 2.2. Experimental field design

The Lahn/Cham1 population trial consisted of 112 recombinant inbred lines (RILs) and the two parents. For the experimental design, we used the augmented design (AD), according to Federer (1956) and Kehel et al. (2010), with 5 checks (Omrabi5, Haurani, Korifla, Waha, and Gidara2). The AD is based on randomized complete block design (RCBD) with 6 blocks. Each trial had 144 plots arranged as a grid layout of 12 rows by 12 columns. The Lahn/Cham1 population was grown in the field for grain yield (GY) evaluation in contrasting environments (Nachit and Elouafi, 2004; Nachit et al., 2016). The GY is harvested from 5 m^2^ plot and estimated as the harvested grain’s weight in kilogram per hectare. The testing sites description is shown in Table 1 with their locations, growing seasons, crop rotations, supplementary irrigation, and temperatures during the trial’s tillering and flowering stages.

**Table 1:** Testing sites, environments (crop rotation) with growing seasons, sowing dates, annual rainfall, added irrigations, and mean temperatures at tillering and flowering stages for the field trials of Lahn/Cham1 population.

Sites	Environment	Season	Sowing date	Rainfall (mm)	Irrigation (mm)	Mean temperature (°C) at	
						Tillering(Zadoks scale 29)	Flowering(Zadoks scale 69)
Tel Hadya/Syria (ICARDA main station) (36°0.1′Nl 36°56′Em, 284m)	02EPa (lentil)	01/02	Mid-October	330	70 at sowing	2	20
	01IRb (vetch above biomass harvested)	00/01	Mid-November	330	100 (50 at early tillering & 50 at booting)	8	24
	01INCc (vetch biomass incorporated in soil)	00/01	Idem	330	None	8	24
	02INCd (vetch biomass incorporated in soil)	01/02	Idem	330	None	8	24
	02RFe (vetch above biomass harvested)	01/02	Idem	330	None	8	24
Breda/Syria (dry) (35°0′Nl 38°0′Em, 300m)	02BRf (fallow)	01/02	Idem	260	None	6	27
Ghab/Syria (high rainfall)(35°38′N 36°14′Em, 280m)	01GHg (faba beans)	00/01	Idem	650	None	5	20
	02GHh (faba beans)	01/02	Idem	614	None	5	20
Raqqa/Syria (irrigated) (35°57′ Nl 39°0′Em, 295m)	01RQi (chickpea)	00/01	Idem	150	450 (150 at sowing, 150 at tillering & 150 at flowering)	10	30
	02RQj (chickpea)	01/02	Idem	150	Idem	10	30
Terbol/Lebanon (high & favorable rainfall) (33°49′Nl 35°58′Em, 890m)	01TRk (faba beans)	00/01	Idem	550	None	2	20

### 2.3. Population genotyping

The genotyping of the population was conducted at the ICARDA durum molecular breeding laboratory. The Lahn/Cham1 population was genotyped with SSR markers and 1500 polymorphic SNPs selected from 9400 SNP platform. The protocol for DNA extraction, molecular assay for microsatellites (SSR), AFLP, and seed storage proteins was conducted as described by Nachit et al. (2001). The genotyping by sequencing of RILs was performed using a whole-genome profiling service for SNP and diversity array technology sequencing (DarTseq** markers). **One hundred microliters of 50 ng µL^-1^ was analyzed using Diversity Array Technology1http://www.diversityarrays.com, as described by Akbari et al. (2006).

### 2.4. Genetic linkage map construction

The genetic linkage map was constructed with 247 molecular markers: 216 SSR, 14 expressed sequence repeats (EST-SSR), 10 AFLP, 6 seed storage proteins (gliadin (Gli) and glutenin (Glu)), and 1 Pseudogliadine gene. Furthermore, we enriched the SSR map with 1425 polymorphic SNPs (Su et al., 2018); the remaining 75 SNPs were unlinked. We used the QTL IciMapping software V 4.1.0.0 (Wang et al., 2016) for mapping. Kosambi mapping function (Kosambi, 1944) was used to transform the recombination frequencies into centiMorgan (cM) distances.

### 2.5. Grain yield QTL detection 

QTL IciMapping software V 4.1.0.0 (Wang et al., 2016) was used to detect the QTLs; the scanning was performed by the model “Inclusive Composite Interval Mapping Additive (ICIM-ADD)” through stepwise regression according to Li et al. (2007). The walking speed for all QTLs was 1.0 cM, and the stepwise regression probability was 0.01. We used a LOD threshold of 3 with 1000 permutations and an error of 0.05. Combined QTL analysis across all environments was conducted to identify QTLs with additive-by-environment (A by E) interaction effects, using the same parameters and the threshold of LOD 3 (Li et al., 2007) for the ICIM method of QTL analysis.

## 3. Results

### 3.1. Trial performance across the contrasting environments

Table 2 shows RIL mean, maximum, and minimum yields along the population parents Lahn (parent 1) and Cham1 (parent 2). In the irrigated and favorable environments, the parent’s grain yield performance is above the mean grain yields of the RILs mean. In the average yielding environments of 4000–5000 kg/ha (02RF, 02INC) and low yielding environments of 2000–2500 kg/ha (02BR), the parent’s grain yield was approximately similar to that of the mean grain yields of the RILs. Table 2 shows statistical parameters, including the trial heritability values. Furthermore, in all environments, several RILs have outyielded both parents (Table 2) significantly.

**Table:** Table 2. Statistical parameters of grain yield (kg/ha) for RIL mean, maximum, and minimum yielders (kg/ha), and parents across testing environment.

Parameter	02RF	01IR	02EP	01INC	02INC	02BR	01GH	02GH	01RQ	02RQ	01TR
RILa mean	4301	8590	8014	6010	5033	2311	6669	7947	6644	7517	6221
Maximum	5257	11217	13753	8457	6563	3373	11033	11933	10133	11083	8667
Minimum	2697	5033	3827	3433	2865	1273	2333	3100	1557	4277	1333
P1b (Lahn)	2873	9825	8787	6363	4821	2470	7517	8600	7867	8100	6000
P2c (Cham1)	3137	9617	10340	6433	5385	2170	8317	9867	7667	8433	7333
LSDd (5%)	436	758	1263	600	424	324	1306	2254	1897	1273	1620
CVe %	8.28	8.15	14.22	8.69	7.2	11.15	16.9	22.85	20.87	13.69	23.54
Trial H2f	0.36	0.43	0.54	0.46	0.32	0.44	0.29	0	0.09	0.31	0
EGAg (%)	5.27	27.39	28.27	19.95	34.1	13.6	40.44	12.19	-6.55	27.59	16.77

The highest RIL grain yield means were achieved in the irrigated environments (01IR), the early sowing environment with supplementary irrigation (02EP), and the favorable and high-input environment (02GH). The maximum yields were observed in the early sowing environment with 13,753 kg/ha (02EP) followed by the favorable/high-input environment with fertile soil (02GH) 11,933 kg/ha, the irrigated environment (01IR) with 11,217 kg/ha, the favorable and high-input environment with fertile soil (01GH) 11,033 kg/ha, and the hot irrigated environment with 11,083 and 10,133 kg/ha (02RQ and 01RQ). The Ghab site with high soil fertility is located in a valley in north-western Syria, where soil is loamy and consists of sand, clay, silt, and black topsoil. Its fertility provides consistent high crops yields across years.

### 3.2. Map construction

The SNPs were mapped to all 14 chromosomes of durum wheat. The A chromosomes had 2902.61 cM (47.41%), whereas the B chromosomes had 3219.61 cM (52.59%). Furthermore, the A chromosomes were covered by 117 SSR markers (47.36%) and 598 SNPs (41.96%) with an average distance between adjacent loci of 4.06 cM. B chromosomes were covered by 130 SSR markers (52.64%) and 827 SNPs (58.04%) with an average distance between adjacent loci of 3.36 cM. The highest number of SNPs mapped was on 2B with 205 SNPs, whereas the lowest number of molecular markers mapped was on 7B with 23 SNP markers (Table 3).

**Table 3:** Assignment and distribution of molecular markers, %, cM size, and coverage across the 14 A and B genomes for SSR and SNP enriched maps in the durum Lahn/Cham1 population.

Chromosome	Markers				Size (cMb) in SSRc/SNPa enriched map	cMb/marker in SSRc/SNPa enriched map
	SSRc	SNPa	All markers	%		
1A	16	85	101	6.04	184.29/320.6	11.52/3.17
2A	19	133	152	9.09	227.73/502.84	11.98/3.31
3A	6	94	100	5.98	85.75/327.11	14.29/3.27
4A	18	110	128	7.66	158.80/474.76	8.22/3.71
5A	13	24	37	2.21	65.18/151.31	5.01/4.09
6A	32	64	96	5.74	481.43/746.24	13.07/7.77
7A	13	88	101	6.04	224.69/379.53	17.28/3.76
A chromosomes	117	598	715	42.76	1427.87/2902.61	12.20/4.06
1B	37	166	203	12.14	252.27/678.09	6.82/3.34
2B	21	205	226	13.52	246.29/694.15	11.73/3.07
3B	11	27	38	2.27	81.13/245.37	7.37/6.46
4B	18	138	156	9.33	111.63/477.61	6.20/3.06
5B	12	166	178	10.65	101.55/609.17	8.46/3.42
6B	21	102	123	7.36	165.68/350.54	7.89/2.85
7B	10	23	33	1.97	105.32/164.68	10.53/4.99
B chromosomes	130	827	957	57.24	1063.87/3219.61	8.18/3.36
Total	247	1425	1672	100	2491.74/6122.22	10.09/3.66

For the group of A chromosomes, 2A harbored the largest number of markers (152) with the highest SNP mapping number (133), followed by 4A (128) with 110 SNPs and by 1A and 7A (101 each) with 85 and 88 SNPs, respectively (Table 3). The 2A showed the highest markers enrichment in the group of A chromosomes with a size of 502.84 cM and a density of 3.31 cM per marker. However, the 6A was the largest chromosome with 746.24 cM, but it was covered only with 96 markers; consequently, it has the lowest cM density per marker (7.77 cM/marker). The least enriched chromosome in the A chromosomes with SNP markers was the chromosome 5A (24 SNP). The entire A chromosomes had 715 markers with a map size of 2902.61 cM and an average density of 4.06 cM/marker (Table 3). 

As for the group of B chromosomes, 2B had the highest number of molecular markers (226). It also had the highest number of SNPs (205) mapped; 1B and 5B followed it with 203 and 178, respectively. In both 1B and 5B, 166 SNPs each were mapped (Table 3). The lowest coverage in B chromosomes was the 7B chromosome with SNPs (23). The total number of molecular markers harbored by the B chromosomes was 957. 

In the B chromosomes, 2B covered 694.15 cM, and it was relatively saturated: 3.07 cM per marker. Moreover, it was the highest enriched genome with SNP markers of the entire genome. The 6B was the most densely saturated chromosome: 2.85 cM/marker and 350.54 cM size. Furthermore, the whole B chromosome was covered by 957 markers with a size of 32,19.61 cM and an average density of 3.36 cM per marker. There were more SNPs mapped in the B chromosomes than in the A chromosomes: 827 for B vs. 598 for A chromosomes; and for the total markers mapped: 957 for B vs. 715 for A chromosomes. The B chromosome map size was larger than that of the A chromosomes: 3219.61 vs. 2902.61 cM. Similarly, for marker density (Table 3), B chromosomes were denser (3.66 cM/marker) than A chromosomes (4.06 cM/marker).

### 3.3. QTL mapping for grain yield in specific environments

In the specific environments, three QTLs were detected in the rainfall-favorable environment (01GH and 02GH), and two in the irrigated and hot environment (02RQ). Furthermore, four QTLs were detected in the cold favorable environment (02EP) and the dry environments (02INC and 02RF).

In 01GH, the first QTL was located at 5A chromosome and flanked on the left by ksm137bp420 and on the right by gwm154bp115 (Table 4). The second QTL was located at 6A and flanked on the left by McagEagc-bp170 and on the right by wsnp_1092345. The last QTL was detected at 6B chromosome, and its left flanking marker was wsnp_1708133, and the right flanking marker was gwm133bp125 (Table 4; Figure).

**Table 4:** Significant QTL regions in contrasting environments for Lahn/Cham1 population with their chromosomal location, position, flanking markers, LOD scores, additive effect, and phenotypic variations.

Environment	Chromosome	Position (cM)	Left marker	Right marker	LODh	PVEi (%)	Addj
01GH	5A	54	ksma137bp420	gwmc154bp115	3.9248	11.4299	450.9197
01GH	6A	508	McagEagcf-bp170	wsnpg_1092345	3.4404	12.1341	520.5310
01GH	6B	14	wsnpg_1708133	gwmc133bp125	3.0045	8.8522	397.7209
02RQ	2A	63	cnlb127bp435	wsnpg_2276833	3.3358	9.435	–518.7356
02RQ	2B	531	wsnpg_1058086	wmce175bp225	4.4356	13.0559	–610.2649
02EP	2A	142	wsnpg_2280363	wmce177bp190	3.2202	12.5648	614.8357
02GH	3B	95	wsnpg_1145565	wsnpg_1005323	3.1525	11.4755	–295.1018
02INC	2A	46	wsnpg_2280371	cnlb127bp435	3.9061	15.0246	–180.9878
02RF	2A	270	barcd183	gwmc312bp189	3.6291	14.1871	–159.9529

**Figure:**
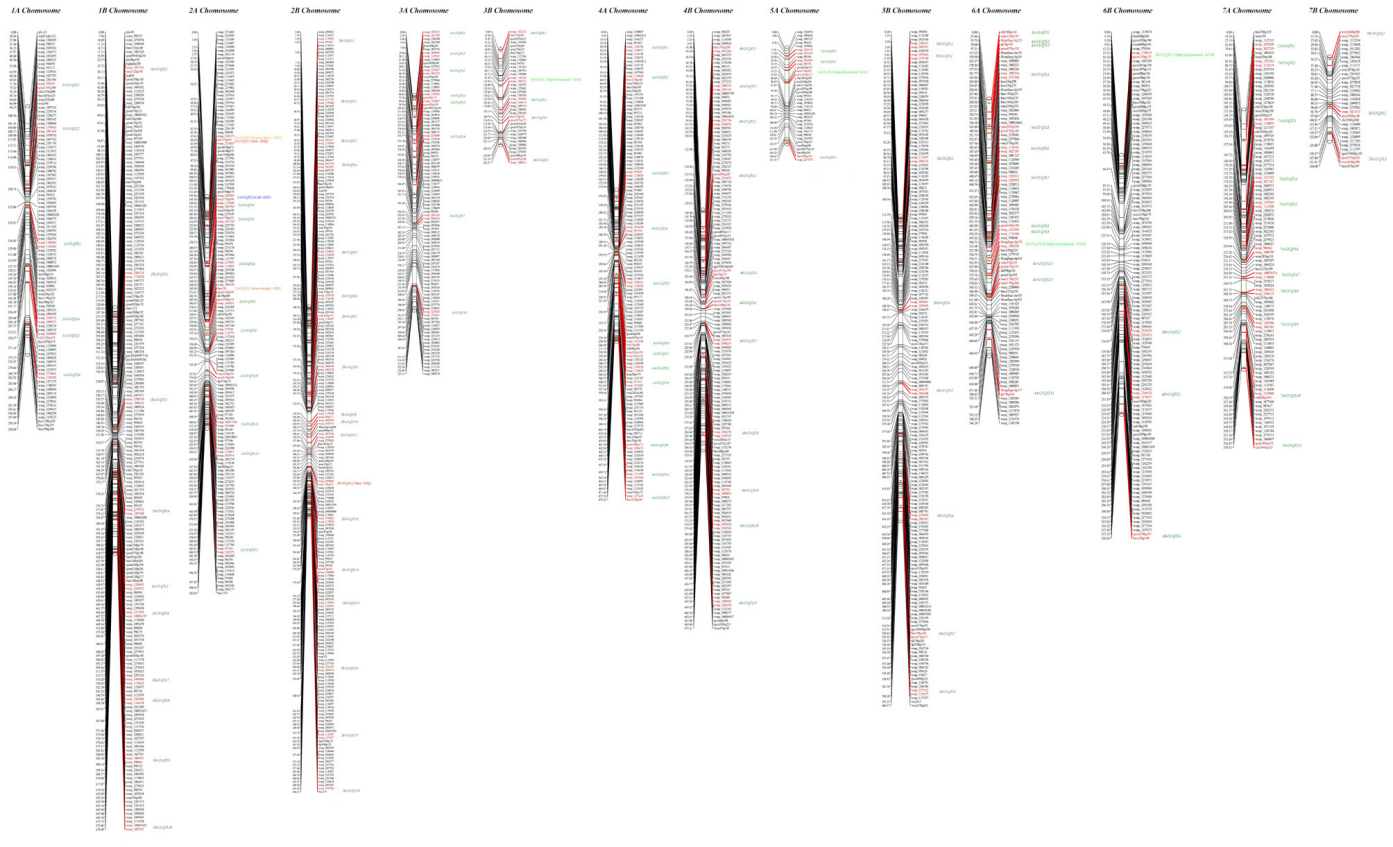
Chromosomes and grain yield QTLs of Lahn/Cham1 genetic linkage map.

In the hot irrigated environment (02RQ), a QTL was detected at 2A with the flanking markers cnl127bp435 and wsnp_2276833 (Table 4; Figure). The analysis also showed another QTL at 2B chromosome, which was flanked by wsnp_1058086 and wmc175p225 (Table 4; Figure).

In addition, four other QTLs were detected, three of which at 2A chromosome in 02EP, 02INC, and in 02RF, whereas the fourth QTL was detected at 3B chromosome in 02GH environment (Table 4; Figure). 

The expression of the QTLs occurred in all environments: moderately stressed (02INC and 02RF), favorable and cold (01GH, 02GH, and 02EP), and hot irrigated (02RQ). The differences of QTL detection between the high-input favorable sites (01GH and 02GH) are probably due to the different seasonal rainfall distribution: 02GH was affected by early drought.

### 3.4. Mapping in multienvironment trials

In multienvironmental mapping, 126 QTLs were detected for GY. The A chromosomes contained 53.17% of the total QTLs detected. The chromosomes 2A, 2B, and 6A harbored the highest QTL numbers: 13, 18, and 13, respectively. The GY-QTL distribution and their flanking markers are reported in Table 5 and Figure.

**Table 5:** Significant QTL regions for multienvironment trials with their chromosomes, position, flanking markers, LOD scores,additive effect, and phenotypic variations.

Chromosome	Position (cM)	Left marker	Right marker	LOD m	LOD (A) n	LOD (AbyE) o	PVE p	PVE (A) q	PVE (AbyE) r	Adds
1A	75	wsnp_993293l	gwm136bp300e	4.25	1.01	3.24	1.91	0.78	1.13	–61.09
1A	105	wsnp_1376461l	wsnp_1091468l	3.45	0.10	3.35	0.91	0.08	0.83	19.37
1A	127	wsnp_1106684l	wsnp_1126484l	5.27	0.36	4.91	2.11	0.29	1.82	37.24
1A	203	wsnp_1093499l	wsnp_2256752l	5.39	0.41	4.98	2.89	0.31	2.57	–38.66
1A	228	wsnp_3020949l	cfa2135bp175h	3.72	0.40	3.32	3.63	0.31	3.33	–38.29
1A	281	wsnp_2276661l	wsnp_1126203l	3.10	0.39	2.71	1.04	0.31	0.74	38.28
1B	26	wsnp_1057112l	ksm112bp290a	3.20	0.24	2.95	0.90	0.19	0.71	30.38
1B	207	wsnp_2266714l	wsnp_1718420l	3.05	0.98	2.07	2.58	0.76	1.82	–60.09
1B	334	wsnp_2280520l	wsnp_1090342l	4.40	0.98	3.43	3.83	0.76	3.07	60.27
1B	379	wsnp_2278332l	wsnp_1207280l	3.04	0.24	2.80	1.72	0.19	1.53	30.22
1B	451	wsnp_1240883l	wsnp_2265852l	6.59	1.84	4.75	3.51	1.45	2.07	84.25
1B	467	wsnp_1215769l	wsnp_100004197l	3.83	0.17	3.66	3.98	0.13	3.85	25.29
1B	528	wsnp_1089860l	wsnp_1138263l	3.09	0.28	2.81	3.49	0.22	3.27	32.32
1B	564	wsnp_2265986l	wsnp_1164138l	3.16	0.03	3.12	1.90	0.02	1.88	10.86
1B	586	wsnp_1006701l	wsnp_998041l	5.27	0.18	5.09	3.36	0.14	3.23	–26.09
1B	677	wsnp_10001692l	wsnp_1092947l	3.46	0.93	2.54	2.59	0.71	1.88	–58.38
2A	46	wsnp_2280371l	cnl127bp435b	8.02	1.73	6.29	4.09	1.35	2.74	–80.09
2A	60	cnl127bp435b	wsnp_2276833l	7.08	4.27	2.81	7.08	2.99	4.09	–119.35
2A	142	wsnp_2280363l	wmc177bp190g	8.38	0.05	8.33	10.59	0.04	10.55	14.45
2A	151	wsnp_1122848l	wsnp_1092763l	7.66	1.33	6.33	7.01	1.03	5.98	–72.61
2A	210	wmc170bp225g	wsnp_1021220l	3.60	1.32	2.28	3.14	1.02	2.12	–69.75
2A	253	wsnp_2278093l	wsnp_1110051l	6.17	2.16	4.02	5.16	1.60	3.56	–87.19
2A	269	wsnp_1000185l	barc183f	7.52	1.84	5.68	3.54	1.41	2.13	–81.99
2A	290	gwm294bp135e	wsnp_1666851l	5.02	1.61	3.40	4.05	1.25	2.81	–77.04
2A	335	wsnp_979520l	wsnp_1128771l	4.24	1.72	2.52	3.07	1.34	1.72	–79.95
2A	377	wmc181bp255g	gwm311bp160e	5.17	2.86	2.31	3.52	1.96	1.56	–97.15
2A	413	wsnp_10017104l	wsnp_1010000l	4.07	0.89	3.18	3.75	0.69	3.06	–57.45
2A	434	wsnp_1128817l	wsnp_3029919l	5.24	2.52	2.72	3.94	1.92	2.02	–95.52
2A	484	wsnp_997103l	wsnp_2281375l	5.24	2.56	2.67	4.83	2.00	2.84	–97.50
2B	4	wsnp_1130302l	wsnp_998518l	3.21	0.16	3.05	3.35	0.12	3.23	23.86
2B	45	wsnp_2257342l	wsnp_1199440l	3.61	0.23	3.38	2.37	0.18	2.19	28.93
2B	61	wsnp_3025617l	wsnp_2278589l	4.08	0.59	3.49	3.73	0.40	3.34	43.44
2B	112	wsnp_1021176l	wsnp_3022997l	5.15	0.16	5.00	4.44	0.12	4.32	24.25
2B	237	wsnp_1204695l	wsnp_1164950l	3.24	0.71	2.53	3.07	0.51	2.56	49.24
2B	282	wsnp_1205720l	wsnp_1160346l	3.63	0.24	3.38	3.49	0.19	3.30	30.38
2B	314	wmc243bp175g	wsnp_1106597l	3.20	0.52	2.68	1.56	0.41	1.16	–45.31
2B	335	wsnp_100140l	wsnp_1082370l	3.09	0.82	2.28	3.79	0.62	3.16	54.40
2B	379	wsnp_1109199l	gwm148bp175e	4.72	0.12	4.59	1.87	0.09	1.78	–21.16
2B	406	wsnp_3025955l	wsnp_1020753l	4.66	0.17	4.49	2.66	0.13	2.53	–24.79
Chromosome	Position (cM)	Left marker	Right marker	LOD m	LOD (A) n	LOD (AbyE) o	PVE p	PVE (A) q	PVE (AbyE) r	Adds
2B	468	wsnp_3027036l	wsnp_1218999l	4.98	1.19	3.79	2.22	0.81	1.41	–63.13
2B	531	wsnp_1058086l	wmc175bp225g	8.08	2.15	5.94	8.17	1.67	6.50	–89.23
2B	552	wsnp_1700082l	wsnp_1179226l	4.80	0.20	4.59	2.27	0.15	2.12	–26.95
2B	575	gwm47bp144e	wsnp_1386000l	4.23	0.45	3.78	2.00	0.36	1.64	–42.25
2B	594	wsnp_1133994l	wsnp_1228193l	3.73	1.00	2.73	2.11	0.75	1.35	–60.56
2B	631	wsnp_2282293l	wsnp_1009576l	5.87	0.62	5.26	3.18	0.49	2.69	–52.58
2B	653	wsnp_1122903l	wsnp_1227557l	3.15	0.10	3.04	1.79	0.08	1.70	–20.49
2B	694	wsnp_1393706l	barc159f	3.63	0.01	3.62	3.17	0.01	3.16	–4.94
3A	0	wsnp_1072225l	wsnp_1667169l	6.41	0.00	6.41	5.50	0.00	5.50	1.63
3A	20	wsnp_1009844l	wsnp_1107433l	3.79	0.21	3.58	3.18	0.16	3.02	27.88
3A	46	wsnp_1232627l	wsnp_3022183l	3.72	0.13	3.60	3.37	0.10	3.28	21.62
3A	67	wsnp_1109262l	gwm5bp178e	4.22	0.03	4.19	2.02	0.03	1.99	–10.92
3A	94	wsnp_1218407l	gwm68bp190e	4.65	1.85	2.79	3.80	1.42	2.38	82.33
3A	151	wsnp_2276600l	wsnp_1220348l	4.13	0.67	3.46	3.09	0.52	2.57	49.95
3A	196	wsnp_1081242l	wsnp_3026254l	5.35	1.26	4.10	4.79	0.97	3.82	69.19
3A	291	wsnp_1237635l	wsnp_1265491l	3.10	0.60	2.50	2.97	0.47	2.50	47.71
3B	1	wsnp_1022121l	barc73bp205f	3.69	0.04	3.65	3.61	0.03	3.58	11.79
3B	92	wsnp_1145565l	wsnp_1005323l	4.48	0.31	4.17	2.56	0.23	2.33	–33.34
3B	164	wsnp_1698409l	wsnp_1698619l	5.16	1.63	3.53	2.49	1.17	1.31	76.47
3B	213	barc77bp220f	gwm547bp175e	3.66	0.92	2.74	2.32	0.67	1.65	57.24
3B	245	gwm582bp200e	wsnp_1088815l	4.15	0.87	3.28	3.07	0.67	2.40	56.77
4A	21	wsnp_1206766l	wsnp_1090457l	3.30	0.00	3.29	2.35	0.00	2.35	4.06
4A	59	wsnp_1130454l	barc113bp140f	7.38	1.17	6.21	3.27	0.86	2.41	66.30
4A	150	wsnp_979658l	wsnp_1218818l	3.48	0.02	3.46	3.21	0.01	3.20	–8.17
4A	186	wsnp_1064430l	wsnp_985258l	3.25	0.21	3.04	4.00	0.16	3.83	–28.01
4A	204	wsnp_1108161l	wsnp_1109226l	3.33	0.45	2.87	1.84	0.33	1.51	–40.10
4A	266	wsnp_1073246l	cfd31bp200i	4.93	0.01	4.92	4.40	0.01	4.39	7.27
4A	276	wmc262bp150g	wmc232bp140g	4.75	0.01	4.74	4.63	0.01	4.62	–7.78
4A	293	wsnp_1309558l	wsnp_1766622l	4.95	0.00	4.95	2.49	0.00	2.49	1.74
4A	321	wsnp_977411l	wsnp_3023881l	4.99	0.00	4.99	6.62	0.00	6.62	3.57
4A	442	gwm160bp172e	wsnp_1086275l	6.34	0.04	6.31	7.30	0.02	7.28	10.36
4A	460	wsnp_1111699l	wsnp_1053664l	5.07	0.00	5.07	5.04	0.00	5.04	0.30
4A	474	wsnp_1073442l	barc327bp245f	5.62	0.19	5.43	7.89	0.13	7.75	25.31
4B	32	barc193bp260	wsnp_1033286	6.48	1.68	4.80	2.70	1.32	1.39	79.35
4B	58	wsnp_1009219	wsnp_1201110	6.33	1.75	4.58	3.99	1.34	2.65	80.30
4B	113	wsnp_2281754	wsnp_1260076	5.22	1.00	4.22	2.03	0.74	1.29	59.50
4B	126	gwm368bp287	wsnp_2254072	6.04	0.15	5.89	3.63	0.12	3.51	23.89
4B	182	gwm513bp190	dp23bp235	4.86	0.11	4.75	2.51	0.08	2.43	20.00
4B	196	gwm513bp150	barc340bp210	4.05	0.06	3.99	2.81	0.05	2.76	15.13
4B	225	wsnp_1264915	wsnp_1090257	3.09	0.02	3.06	1.93	0.02	1.91	–9.34
Chromosome	Position (cM)	Left marker	Right marker	LOD m	LOD (A) n	LOD (AbyE) o	PVE p	PVE (A) q	PVE (AbyE) r	Adds
4B	290	wsnp_1036270	wsnp_1220743	3.85	0.24	3.61	3.13	0.17	2.96	–28.40
4B	336	wsnp_992780	wsnp_1090893	3.80	0.15	3.65	1.68	0.11	1.57	22.84
4B	375	wsnp_1050158	wsnp_1010596	3.01	1.45	1.55	1.67	1.10	0.57	72.61
4B	449	wsnp_1206903	wsnp_1204109	3.21	0.82	2.40	2.25	0.62	1.62	–55.05
5A	12	wsnp_2283193l	wsnp_1071495l	3.80	0.02	3.78	2.38	0.02	2.36	9.03
5A	35	wsnp_3022906l	wsnp_981078l	4.25	0.00	4.25	1.48	0.00	1.48	1.08
5A	55	ksm137bp420a	gwm154bp115e	8.60	3.33	5.27	6.37	2.62	3.75	111.84
5A	148	barc100bp110f	wsnp_2257379l	3.70	2.10	1.60	3.42	1.56	1.87	86.39
5B	3	wsnp_2280241l	wsnp_1095057l	5.21	2.65	2.56	3.63	2.06	1.57	–99.43
5B	10	wsnp_1079002l	wsnp_2279190l	6.93	3.72	3.21	4.00	2.83	1.16	–116.00
5B	89	wsnp_1111097l	wsnp_1094116l	3.42	0.03	3.40	3.50	0.02	3.48	–9.82
5B	187	wsnp_1695891l	wsnp_1078695l	4.23	0.19	4.04	4.90	0.14	4.76	–26.03
5B	332	wsnp_1091357l	wsnp_3024240l	3.41	0.08	3.33	2.83	0.06	2.77	–17.13
5B	434	wsnp_2278459l	wsnp_1081891l	3.39	0.59	2.79	3.61	0.47	3.14	–47.05
5B	521	barc74bp120f	gwm371bp317e	3.03	0.58	2.45	2.12	0.44	1.68	–45.84
5B	584	wsnp_2277122l	wsnp_1126527l	3.67	0.54	3.13	3.41	0.42	3.00	44.54
6A	35	cfd190bp160i	wmc123bp135g	3.70	0.39	3.30	2.58	0.29	2.29	45.50
6A	56	McacEaac-bp225d	pk7bp190k	4.66	2.51	2.15	4.28	1.93	2.35	108.11
6A	97	pk7bp190k	gwm497bp118e	3.05	0.76	2.29	1.42	0.58	0.84	–58.32
6A	185	wsnp_1002536l	wsnp_2331540l	3.65	0.30	3.35	3.00	0.23	2.77	34.26
6A	345	wmc201bp250g	gwm570bp140e	4.32	0.50	3.82	1.75	0.40	1.35	43.80
6A	387	wsnp_1126162l	wsnp_30272001l	3.98	0.24	3.74	3.84	0.18	3.66	29.25
6A	401	wsnp_1202412l	wsnp_1202955l	3.81	0.02	3.79	4.07	0.02	4.05	–9.48
6A	443	gwm169bp190e	wsnp_1231938l	5.39	0.67	4.72	3.29	0.49	2.80	48.34
6A	477	wsnp_1231938l	wsnp_1125460l	4.09	0.06	4.03	2.25	0.05	2.20	–14.68
6A	507	McagEagc-bp170c	wsnp_1092345l	6.00	0.23	5.76	4.33	0.18	4.15	33.96
6A	566	wmc41bp155g	gwm617bp123e	5.25	2.01	3.23	2.55	1.40	1.15	–112.97
6A	578	wmc173bp235g	wmc175bp260g	4.80	1.26	3.54	3.75	0.90	2.85	–122.39
6A	700	McagEagc-bp117c	pk19bp160k	3.66	0.05	3.61	2.66	0.04	2.62	14.80
6B	15	wsnp_1708133l	gwm133bp125e	7.26	0.69	6.57	5.59	0.49	5.09	48.87
6B	175	wsnp_2329478l	wsnp_2261831l	4.85	0.00	4.84	1.95	0.00	1.95	4.27
6B	201	wsnp_2266428l	wsnp_1178437l	3.84	0.19	3.66	2.45	0.14	2.31	25.77
6B	350	gwm219bp255e	barc24bp190f	3.35	0.43	2.93	1.94	0.32	1.62	39.68
7A	43	wsnp_1070109l	wsnp_3027359l	4.09	0.37	3.72	3.69	0.28	3.41	36.34
7A	88	wsnp_1225184l	wsnp_1210139l	3.81	0.20	3.61	2.69	0.15	2.53	27.06
7A	150	wsnp_1041994l	wsnp_1128093l	5.42	0.54	4.88	4.18	0.34	3.84	40.19
7A	177	wsnp_1221352l	wsnp_3027187l	6.82	1.03	5.79	6.02	0.76	5.25	60.38
7A	202	wsnp_1197565l	wsnp_1125508l	3.78	1.48	2.30	2.73	1.13	1.60	73.22
7A	223	wsnp_996562l	wsnp_1080709l	4.23	1.35	2.88	4.23	1.06	3.17	71.05
7A	244	wsnp_100078174l	wsnp_1130808l	3.31	1.13	2.18	3.55	0.88	2.67	64.83
Chromosome	Position (cM)	Left marker	Right marker	LOD m	LOD (A) n	LOD (AbyE) o	PVE p	PVE (A) q	PVE (AbyE) r	Adds
7A	268	wsnp_1004422l	wsnp_2280119l	4.34	0.03	4.31	5.06	0.02	5.04	–10.06
7A	289	wsnp_1262886l	wsnp_1001581l	3.80	0.00	3.80	2.17	0.01	2.16	7.00
7A	326	wsnp_1235900l	cfd020bp305i	3.14	0.01	3.13	2.68	0.01	2.68	5.71
7A	379	gwm344bp135e	cfa2040bp325h	4.90	0.23	4.67	5.82	0.17	5.65	–28.56
7B	5	wmc606bp200g	barc279bp205f	4.34	0.32	4.02	3.13	0.20	2.94	30.70
7B	93	wsnp_3021533l	gwm400bp148e	4.40	0.03	4.37	2.80	0.02	2.77	10.66
7B	150	gwm573bp220e	wmc426bp200g	5.56	0.96	4.60	3.03	0.69	2.35	57.47

aksm: Kansas State University (EST-SSR); bcnl: Cornell University (EST-SSR); cMcagEagc: MseI+cag and EcoRI+agc (AFLP); dMcacEaac: MseI+cac and EcoRI+aac (AFLP); egwm: Gatersleben Wheat Microsatellite (SSR); fbarc: Beltsville Agriculture Research Center (SSR); gwmc: Wheat Microsatellite Consortium ; hcfa: Clermont-Ferrand A-genome (SSR); icfd: Clermont-Ferrand D-genome (SSR); jdp: Dehydrin primers “functional genes” (SSR); kpk: PK Gupta /india (SSR); lwsnp: Wheat SNP (SNP); mLOD: Logarithm of odds; nLOD(A): LOD score for additive and dominance effects; oLOD(AbyE): LOD score for additive and dominance by environment effects; pPVE: Phenotypic variation explained by QTL at the current scanning position; qPVE(A): Phenotypic variation explained by additive and dominance effect at the current scanning position; rPVE(AbyE): Phenotypic variation explained by additive and dominance by environment effect at the current scanning position; sAdd: Additive effect at the current scanning position; positive numbers shows allelic effect from Lahn, negative numbers from Cham1.

The number of QTL contributions to grain yield was 126. The QTL detected covered all chromosomes. Furthermore, the A chromosomes QTL contributions were higher than in B chromosomes 67 vs. 59. The chromosomes with the highest number of QTL were 2B (18) followed by 2A and 6A (13) for each, whereas the lowest chromosome with QTL was 7B (Table 5; Figure). The comparison with several published wheat mapping populations (Table 6) showed that Lahn/Cham1 population harbored more QTLs contributing to grain yield and in all A and B chromosomes (LOD used for QTL detection was ≥3). Some chromosomes (Table 5; Figure) were harboring a large number of QTLs, as in the case of 1A, 1B, 2A, 2B, 4A, 6A, and 7A. 

Moreover, the QTLs with an important QTL*Environment effects were on the chromosomes 4A, where the additive effects ranged from 25.31 to 66.3 and from –40.1 to –28.0. On 5B, the additive effect ranged from –116 to –26.03, and one QTL with an additive effect of 44.54. On 7A, several QTLs with a positive additive effect ranged from 27.06 to 73.22 and one QTL with a negative additive effect (–28.56). The contribution of the Lahn parent was 74 QTLs, of which 40 originated from A chromosomes and 34 from B chromosomes, while Cham1 contributed with 52 QTLs, 29 of which were originated from A chromosomes and 23 from B chromosomes.

## 3. Discussion

The Lahn/Cham1 genetic linkage map has been shown to share several SSR markers with other linkage maps (Nachit et al., 2001; Elouafi and Nachit, 2004; Haile et al., 2012; Zhang et al., 2012; Zhang et al., 2012; Nagel et al., 2014 Alsaleh et al., 2015). It shared an important number of SSR markers with the high-density microsatellite consensus maps for bread wheat (Somers et al., 2004) and durum wheat (Maccaferri et al., 2015). Moreover, Lahn/Cham1 map was concordant with the marker orders of the consensus maps.

Su et al. (2018) adopted a similar approach to include SNP markers. Similarly, Zeng et al. (2019) constructed a bread wheat linkage map with SNP markers using the Wheat 35K SNP array for genotyping the RILs of the population MX169 X CW86.

Kirigwi et al. (2007) reported that molecular markers on chromosome 4A linked to grain yield, explaining 20% of the phenotypic variation of grain yield. Concerning QTL detection, grain yield QTLs in wheat have been described in various studies (Maccaferri et al., 2010; McIntyre et al., 2010; Soriano et al., 2017). In Kuchel et al. (2007), bread wheat linkage map, which was mainly based on microsatellite molecular markers, the QTLs linked with grain yield were detected on chromosomes1B, 2D, 3B, 5A, 6B, 7A, 7B, 7D. Multiple QTL detection on similar chromosomes were also reported by Bogard et al. (2011). Additionally, Maccaferri et al. (2008) also reported among the 16 QTLs that affected grain yield, two major QTLs on chromosomes 2BL and 3BS showed significant effects across several environments. Kato et al. (2000) also recovered five regions in wheat chromosome 5A that contributed to grain yield; and according to Heidari et al. (2011), QTLs located on chromosomes 6A, 6B, and 6D accounted mostly for total grain yield variation in wheat. Furthermore, McIntyre et al. (2010) established that several chromosomes harbored QTLs for grain yield on chromosomes 1B, 1D, 4A, 4D, 6A-a, 6B, and 7A. In addition, Quarrie et al. (2005) found that the strongest grain yield QTL effects were located on chromosomes 7AL and 7BL. Under drought conditions, the microsatellite wmc89 located on 4A chromosome was suggested as a marker to enhance drought tolerance in wheat (Kirigwi et al., 2007). Furthermore, Bennett et al. (2012) found that two of the detected QTLs linked to heat tolerance were located on chromosome 3B.

 Marza et al. (2006) showed that the QTL contribution effects ranged from 7% to 23% in Ning7840xClark wheat population, where the marker alleles from the parent Clark were associated with a positive effect for the majority of QTLs for yield and its components on 1AL, 1B, 4B, 5A, 6A, and 7A, and specific QTLs on 2BL, 2BS, 2DL, and 6B. Tura et al. (2019) identified 38 grain yield QTLs spread over the whole genome of the Excalibur/Kuri population of bread wheat with the exception of 5D and 6A chromosomes. The grain yield QTLs with the highest QTL*Environment effects were observed on chromosomes 4A, 5B, and 7A. In the Lahn/Chm1 population, the QTLs linked to grain yield were harbored in all 14 chromosomes. These results agree with those of other studies (Heidari et al., 2011; Zhang et al., 2012; Farré et al., 2016). These results were also similar to those of the recently published work by Tura et al. (2019). In contrast, most of the populations showed a few QTLs (Table 6). It appears that the choice of genetically diverse population parents is of paramount importance to detect all involved QTLs linked with wheat grain yield (Table 6).

**Table 6:** Comparison of detected grain yield QTLs harbored by A and B chromosomes in different wheat mapping populations.

	Kuchel et al. (2007)	Maccaferri et al. (2008)	Bennett et al. (2012)	Kirigwi et al. (2007)	Heidari et al. (2011)	Quarrie et al. (2005)	Bogard et al. (2011)	McIntyre et al. (2010)	Marza et al. (2006)	Tura et al. (2019)	Lahn/Cham1 population			
												Parent QTLs contribution per chromosome		
RIL/Marker	182/251	249/254	250/456	127/ 136	107/371	96/567	140/499	192/587	132/410	155/3502	112/1672	TotalQTL	Lahn	Cham1
1A									+	+	+	6	3	3
1B	+							+	+	+	+	10	7	3
2A										+	+	13	1	12
2B		+							+	+	+	18	7	11
3A			+							+	+	8	7	1
3B		+	+				+			+	+	5	4	1
4A			+	+				+		+	+	12	8	4
4B									+	+	+	11	8	3
5A							+		+	+	+	4	4	0
5B			+							+	+	8	1	7
6A					+			+	+		+	13	8	5
6B					+		+	+	+	+	+	4	4	0
7A			+			+	+	+	+	+	+	11	9	2
7B						+	+			+	+	3	3	0

Furthermore, the analysis of the multienvironment trial showed the overall QTLs present on the 4B chromosome has an important additive effect with parent Lahn contribution ranging from 15.13 to 80.30 and the parent Cham1 contribution from –55.05 to –9.34. In light of this, it appears that the 4B chromosome contributes largely to grain yield in the dry areas. 

 Our results under stressed environments showed agreement with those of Ribaut et al. (1997) and Almeida et al. (2013). It is expected that using marker-assisted selection for grain yield under drought and heat stresses will improve breeding under stress conditions. 

Several studies have indicated the presence of QTLs and candidate genes involved in response to drought in the 4B chromosome (Nachit and Elouafi, 2004; Habash et al., 2009). In addition, in Mediterranean terminal stress, drought is usually combined with heat stress; both stresses are a major factor limiting grain yield in the Mediterranean region (Nachit, 1992; Nachit and Elouafi, 2004). Other findings on 4B have revealed the presence of QTLs linked to total biomass yield, grain yield, and straw yield (Li et al., 2014). Recently, the International Wheat Genome Sequence (IWGSC) released a wheat genome reference sequence along with annotated genes (called RefSeq v.1.0, IWGSC 2018)Wheat@URGI. Seq Repository [Online]. Website http://wheaturgi.versailles.inra.fr/Seq-Repository [Accessed 22 Jan 2021]. This information, along the whole genome sequence dataset of 16 wheat varieties (Edwards et al., 2012), allows identifying new SNP markers for QTL fine-mapping and identifying genes responsible for abiotic and biotic stress tolerance in wheat; as consequence, SNP markers linked to detected QTLs need to be annotated for genes involved in traits tolerance/resistance and grain yield performance in diverse and contrasting environments.
